# Improvement of Mercuric Chloride-Induced Testis Injuries and Sperm Quality Deteriorations by *Spirulina platensis* in Rats

**DOI:** 10.1371/journal.pone.0059177

**Published:** 2013-03-28

**Authors:** Gaber E. El-Desoky, Samir A. Bashandy, Ibrahim M. Alhazza, Zeid A. Al-Othman, Mourad A. M. Aboul-Soud, Kareem Yusuf

**Affiliations:** 1 Chemistry Department, College of Science, King Saud University, Riyadh, Saudi Arabia; 2 Zoology Department, College of Science, King Saud University, Riyadh, Saudi Arabia; 3 Pharmacology Department, National Research Center, Cairo, Egypt; 4 Biochemistry Department, Faculty of Agriculture, Cairo University, Giza, Egypt; 5 Department of Clinical Laboratory Sciences, College of Applied Medical Sciences, King Saud University, Riyadh, Saudi Arabia; National Cancer Institute, United States of America

## Abstract

The present study was undertaken to investigate the protective effect of the filamentous cyanobacterium *Spirulina platensis* (*S. platensis*) on mercury (II) chloride (HgCl_2_)-induced oxidative damages and histopathological alterations in the testis of Wistar albino rats. The animals were divided into four equal groups, ***i*** *)* control, ***ii*** *)* HgCl_2_, ***iii*** *) S. platensis* and ***iv*** *)* combination of HgCl_2_+*S. platensis*. Oxidative stress, induced by a single dose of HgCl_2_ (5 mg/kg, bw; subcutaneously, s.c.), substantially decreased (*P*<0.01) the activity level of testicular key enzymatic antioxidant biomarkers (superoxide dismutase, SOD; catalase, CAT and glutathione peroxidase, GPx), oxidative stress makers (blood hydroperoxide; testicular reduced glutathione, GSH and malondialdehyde, MDA), and testicular mercury levels. Moreover, HgCl_2_ administration resulted in a significant (*P*<0.01) increase in the number of sperms with abnormal morphology and decrease in epididymal sperm count, motility, plasma testosterone level and testicular cholesterol. Furthermore, HgCl_2_ exposure induced histopathological changes to the testis including morphological alterations of the seminiferous tubules, and degeneration and dissociation of spermatogenic cells. Notably, oral pretreatment of animals with *Spirulina* (300 mg/kg, bw) lowered the extent of the observed HgCl_2_-mediated toxicity, whereby significantly reducing the resulting lipid peroxidation products, mercury accumulation in the testis, histopathological changes of the testes and spermatozoal abnormalities. In parallel, the pretreatment with *Spirulina* also completely reverted the observed Hg-Cl_2_-induced inhibition in enzymatic activities of antioxidant biomarkers (SOD, CAT and GPx) back to control levels. The pretreatment of rats with *S. platensis* significantly recovered the observed HgCl_2_-mediated decrease in the weight of accessory sex organs. Taken together, our findings clearly highlight the role of *S. platensis* as a protective modulator of HgCl_2_-induced testicular injuries and suggest some therapeutic potential in mammals. Further investigation of therapeutic strategies employing *Spirulina* against heavy metals toxicity in humans is therefore warranted.

## Introduction

Heavy metals have become one of many contaminants found in our environment. Many of these metals, including lead, mercury, cobalt, cadmium, and chromium are known to exert toxic effects on testicular function, while others such as zinc, manganese, and selenium have been shown to be essential for normal functioning of the testis [1], [2], [3]. Mercury is a ubiquitous element in the environment causing oxidative stress in the exposed individuals leading to tissue damage. Its contamination and toxicity has posed a serious hazard to human health. The release of mercury from dental amalgam dominates exposure to inorganic mercury and may have an acceptable risk among the general population [4]. Human exposure to mercury can occur by inhalation, ingestion and consumption via food chain. Adverse effects of metals on human reproduction and development continue to be a demanding challenge for researchers. Mercury compounds are known to affect testicular spermatogenic and steroidogenic functions in experimental animals [5]. Oral exposure of mercuric chloride produced adverse effects on the reproductive performance of mice [6]. Mercury affects accessory sex glands function in rats and mice through androgen deficiency [7], [8]. Decrements in sperm count, motility and morphology have been reported in methyl mercury exposed monkeys and rodents [8], [9]. The activity of superoxide dismutase, glutathione peroxidase and glutathione reductase of sperm incubated in mercury decreased while thiobarbituric reactive substances (TBARS) levels and H_2_O_2_ generation were increased [10]. The treatment of rats with Hg led to a decrease in GSH levels in different tissues [11]. Hg induced oxidative stress in the testicular tissue of the rat as manifested by a decrease in SOD and catalase and an increase of malondialdehyde (MDA) levels [12].

Mercuric chloride is one of the most toxic forms of mercury because it easily forms organomercury complexes with proteins [13]. The inorganic ionic mercury has great affinity for SH groups of biomolecules, such as glutathione (GSH) and sulfhydryl proteins, which may contribute to its toxicity [14]. Once bound to GSH, Hg can leave the cell to circulate in serum or lymph and be deposited in other organs or tissues [13]. Mercuric chloride is considered to be one of the pro-oxidants that induce oxidative stress [6]. Oxidative stress occurs when the production of reactive oxygen species (ROS) such as, superoxide anion (•O^−^_2_), hydrogen peroxide (H_2_O_2_) and the hydroxyl radical (•OH) exceeds the body's defense mechanism, causing damage to macromolecules such as DNA, proteins and lipids [15] and trigger many pathological processes in the male reproductive system [16]. There is evidence that ROS may have a detrimental effect on critical components of the steroidogenic pathway [17]. Excessive production of ROS above normal levels results in lipid peroxidation and membrane damage leading to loss of sperm motility [7], inactivation of glycolytic enzymes and damage to the acrosomal membranes [18] which render the sperm cell unable to fertilize the oocyte, or produce a viable pregnancy [19].

*Spirulina platensis*, recently renamed Arthrospira, is a filamentous cyanobacterium (blue-green alga) that belongs to the *Oscillatoraceae* family and has a long history for use as food. It is rich in proteins, lipids, carbohydrates and some vital elements like zinc, magnesium, manganese, selenium, ß-carotene, riboflavin, α-tocopherol and α-linoleic acid [20]. The antioxidant properties of spirulina and its capacity to scavenge hydroxyl radicals [21], and to inhibit lipid peroxidation [22] have attracted the attention of many researches. Spirulina species exhibited various biological activities such as antihypertensive and antihyperlipidemic [23]. Moreover, *Spirullina fusiformis* provides protection against mercuric chloride induced oxidative stress and alteration of antioxidant defense mechanism in the liver [24]. These activities were largely related to phycocyanin, an active protein of Spirullina [25]. It was reported that feeding of mice a diet supplemented with 30% of *Spirulina maxima* for 13 weeks did not produce any toxic effects [26]. Moreover, *Spirulina maxima* treatment was not associated with any adverse effects on reproductive performance, including male and female fertility and duration of gestation in rats [27]. Furthermore, no significant alteration was observed in the motility and shape of sperm of rats treated with *Spirulina maxima* (30%) incorporated into the diet for 5 days [28]. Oral administration of *Spirullina fusiformis* to mice can modulate mercury induced alteration in testicular acid and alkaline phosphatase activities [29]. To our knowledge, there is no available report until now describing the protective properties of *S. platensis* against mercury-induced injuries to male reproductive system.

In the present study, an attempt has been made to evaluate the protective potential of *S. platensis* against mercury-induced oxidative damage to testes of male rats by studying testicular antioxidant defense system, sperm quality and plasma testosterone level.

## Materials and Methods

### Animals

Male Wistar albino rats (*Rattus norvegicus*), weighing 180–200 g, were obtained from the animal house, Faculty of Pharmacy, King Saud University. The animals were housed throughout the experiment in polypropylene cages (each cage housing eight animals) and allowed to acclimatize to laboratory environment for seven days before the beginning of the experiment. Animals were maintained under controlled conditions of temperature (23±1°c), humidity (50±15%) and normal photoperiod (12–12 h light-dark cycles). Rats were allowed free access to standard dry pellet diet and water *ad libitum*. This study was conducted in the Zoology Department, Faculty of Science, King Saud University, Saudi Arabia. The care and handling of experimental animals were carried out according to the animal ethical committee of the College of Pharmacy, King Saud University.

### Test chemicals

Mercury in the form of HgCl_2_ was purchased from Merck (Darmstadt, Germany) (Product no. 104417), while *S. platensis* was obtained from Alibaba Company (China) in the form of tablets. 5–5-dithio-bis(2-nitrobenzoic acid) (DTNB), thiobarbituric acid and reduced glutathione were purchased from Sigma-Aldrich Corp. (St. Louis, MO USA).

### Treatments

The animals were divided into four groups, each containing eight rats. Animals in group I were used as control group and no treatment was given to these rats. Animals in group II were given *S. platensis* daily by gavage for 60 consecutive days at dose level of 300 mg/kg b.wt [30] suspended in water. Animals in group III were administered a single dose (5 mg/kg bw) of mercury (II) chloride (HgCl_2_) by subcutaneous (s.c.) route with the help of Hamilton syringe [31], dissolved in water three-time a week for 60 days. The s.c. route was chosen to ensure the exact and the proper uptake of HgCl_2_. The administered dose of 5 mg/kg bw (i.e., 15 mg/kg bw/wk) has been chosen based on previous studies which employed HgCl_2_ doses of 5, 10 and 20 mg/kg bw/day. These doses were optimal for the induction of testicular histopathological changes testes without observed toxicity [32] Animals in group IV were given *S. platensis* (300 mg/kg bw) by gavage for 10 consecutive days before mercuric chloride administration and continued up to 60 days along with HgCl_2_ treatment.

### Collection of samples

At the end of the experimental period, blood was drawn from the animals by puncturing retro-orbital venous sinus. Whole blood was used for the determination of hydroperoxide level, while separated plasma was used to determine testosterone level. After collection of blood samples, the animals from all groups were autopsied under light ether anesthesia. Subsequently, testes, vas deferens, epididymis, prostate glands and seminal vesicles were excised from surrounding tissues and placed into tube. Thus, organs were dried between two sheets of filter paper and their wet weight was determined. Next, the relative organ weight was calculated by use of the formula: organ weight/body weight ×100. Left epididymis was weighed, while right epididymis was rinsed in warm phosphate buffered saline (PBS) and incubated at 37°C for the evaluation of sperm quality. Whereas, left testes were processed for histological study, right testes were processed for determination of MDA, GSH, SOD, CAT, GPx and cholesterol.

### Biochemical studies

#### Determination of lipid peroxide levels

Blood hydroperoxide level was evaluated using an analytical system (Iram, Parma, Italy). The test is a colorimetric test that takes advantage of the ability of hydroperoxide to generate free radicals after reacting with transitional metals, when buffered chromogenic substance is added; a colored complex appears. This complex was measured spectrophotometrically. Lipid peroxidation level in the testis was measured by a method [33] using thiobarbituric acid reactive substances (TBARS) with some modifications as previously described [34]. Testis was homogenized in ice cold 0.15 M KCl (10%) and the concentration of TBARS was expressed as nmol of MDA per mg protein using 1,1,3,3-tetramethoxypropane as standard. The absorbance was read at 532 nm.

#### Determination of testicular mercury

Total mercury concentration was determined in testes using a previously reported protocol [35]. The samples were heated with 7 ml nitric acid and perchloric acid (2v/1v) in boiling water bath until evaporation. After digestion, the samples were diluted by definite volume of distilled water and filtered. The mercury concentration was measured in samples by use of atomic absorption, Model RA-2 (Tokyo, Japan). Data are presented as total mercury (µg/g of tissue dry).

#### Determination of testicular reduced glutathione (GSH)

Reduced form of glutathione was determined using Ellman's reagent 5-5-dithio-bis (2-nitrobenzoic acid) (DTNB) as a coloring reagent [36]. The absorbance was read at 412 nm by spectrophotometer. GSH concentration was calculated from a standard curve.

#### Determination of testicular superoxide dismutase (SOD)

Testicular superoxide dismutase was assayed by the method of Asada [37], which involves the inhibition of photochemical reduction of nitro blue tetrazolium (NBT) at pH 8.0. A single unit of enzyme is defined as the quantity of superoxide dismutase required to produce 50% inhibition of photochemical reduction of NBT. The absorbance was read at 580 nm against a blank using UV – Vis spectrophotometer. The activity was expressed as U/mg protein.

#### Determination of testicular catalase (CAT)

Catalase activity was estimated in testis homogenate by the method reported by Aebi [38]. The specific activity of catalase has been expressed as µmoles of H_2_O_2_ consumed/min/mg protein. The difference in absorbance at 240 nm per unit time is a measure of catalase activity.

#### Determination of testicular glutathione peroxidase (GPX)

The activity of the antioxidant enzyme glutathione peroxidase was determined using glutathione reductase and NADPH. This method is based on the oxidation of NADPH at 25°C, which is indicated by the decrease in absorbance at 340 nm [39]. Results are expressed in U/mg protein.

#### Determination of testicular cholesterol

The estimation of testicular cholesterol was carried out by the method of Zlatki [40]. To test tubes containing 5 ml of working FeCl_3_ solution, 0.2 ml of testis homogenate prepared in glacial acetic acid was added. The contents were mixed and 3 ml of concentrated H_2_SO_4_. The optical density after color development was read at 540 nm on a spectrophotometer and expressed as mg/100 mg tissue wt.

#### Hormonal assay

Plasma testosterone concentration was measured by enzyme immunoassay using commercial kit from Diagnostic products Co. (Los Angeles, CA USA).

### Sperm analysis

#### Collection and incubation of epididymal sperm

Spermatozoa were obtained from the fresh right epididymis of adult rat described by Narayana [41]. Briefly, epididymis was cut into small pieces with a sharp razor blade and dispersed in 3 ml of phosphate buffered saline (pH 7.2) to obtain a suspension with gentle stirring. Dispersed sperm samples were kept in an incubator.

#### Sperm motility

Ten µl of the suspension was placed in a warm slide and the percentage of motile sperm was counted under Nikon binocular microscope with warmed stage and about 300 spermatozoa were evaluated. Motility was then expressed as the percentage of motile spermatozoa.

#### Sperm count

The suspension was filtered. An aliquot from the suspension (up to 0.5 ml) was taken in leukocyte micropipette of hemocytometer and diluted with phosphate buffered solution up to the mark 11. The suspension was well-mixed and charged into Neubauer's counting chamber. The total sperm count in 8 big squares was determined and multiplied by 3×5×10^4^ [42] to express the number of spermatozoa/epididymis (million/epididymis).

#### Sperm abnormalities

For the evaluation of the sperm morphology the filtrate (obtained as described above in Section 2.7.3) was stained with 1% eosin and morphological defects were analyzed [42]. Briefly, the spermatozoa in the smears were visualized under light microscope (400×) and any abnormalities of either heads or tails were noted. Three hundred sperms were screened for each animal and total abnormality was expressed as percentage of incidence/300 sperm/animal.

### Histological determination

For microscopic evaluation, testis tissues were fixed in 10% formol saline, embedded in paraffin, sectioned at 5 µm and stained with hematoxylin/eosin. Sections were studied under light microscope at 400× magnification.

### Statistical analysis

All values from duplicate measures for each sample (n = 8) were expressed as means with ± S.E. Statistical analysis of data was performed using two-way ANOVA followed by Fisher's least significant difference (LSD) procedure for comparison of various treatments using the IBM SPSS software (version 20.0, SPSS Inc., Chicago, IL). Difference in statistics at *P*<0.05 were considered significant.

## Results

### Lipid peroxidation products and testicular mercury concentration

Results presented in Table 1 indicate that there was a statistically significant decrease (P<0.01) in the level of blood hydroperoxide in *Spirulina*-treated animals (Group II), while the reduction in MDA level was not significant. Mercuric chloride (HgCl_2_) administration resulted in a significant (P<0.01) elevation in the levels of blood hydroperoxide and testicular MDA by 2.15 and 1.36 folds, respectively, as compared to values in the control group I. The blood hydroperoxide level also increased significantly (P<0.01) in the combined treatment of *S. platensis* with HgCl_2_ by 1.60 fold, while testicular MDA did not change significantly compared to the control values. Moreover, the hydroperoxide or MDA level of *S. platensis*+HgCl_2_ group was significantly (P<0.01) lower than that of exposed to HgCl_2_ alone. Although, mercury level in testis exhibited a significant elevation (P<0.01) in both HgCl_2_ and *S. platensis*+HgCl_2_ groups; its level in the later group was significantly less than that of the HgCl_2_ group.

**Table 1:** Effect of *S. platensis* on lipid peroxidation products and testicular mercury concentration in experimental HgCl_2_-exposed rats as compared to control group.

Groups				
*Spirulina*+HgCl_2_	HgCl_2_	*Spirulina*	Control	Parameters
45.96±2.89^**b^	61.23±5.15^**^	24.11±0.32^**^	28.42±0.63	Blood hydroperoxide level (mg/100 ml)
5.20±0.05^b^	6.96±0.05^**^	4.81±0.13	5.10±0.06	Testicular MDA (nmol/mg protein)
1.40±0.02^**b^	2.15±0.08^**^	00.00	00.00	Mercury concentration (µg/g dry tissue)

The relative organ weight was calculated by use of the formula: organ weight/body weight ×100. Each value represents the mean ± S.E., n = 8. Values marked with an asterisk (*) differ significantly from control value (** P <0.01), while those marked with letter differ significantly from HgCl_2_ group (P<0.01).

### Testicular reduced glutathione and enzymatic antioxidant biomarkers

A significant (P<0.01) increase in reduced glutathione (GSH) and SOD (P<0.05) was observed in *Spirulina* group (Table 2). Although HgCl_2_ exposure alone resulted in a significant (P<0.01) inhibition in the level of testicular GSH and activity levels of testicular antioxidant enzymes (SOD, CAT and GPx,) as compared to the control group, *S. platensis* administration to HgCl_2_-exposed rats prevented the observed decrease of these parameters.

**Table 2:** Effect of *S. platensis* on testicular glutathione content and activities of antioxidant biomarker enzymes in experimental HgCl_2_-exposed rats as compared to control group.

Groups				
*Spirulina*+HgCl_2_	HgCl_2_	*Spirulina*	Control	Parameters
338.23±2.50^b^	231.92±1.23^**^	374.12±2.50^**^	342.48±2.05	GSH (nmol/mg protein)
0.83±0.007^b^	0.56±0.01^**^	0.84±0.01	0.85±0.01	GPX (µ/mg protein)
5.59±0.11^b^	3.63±0.09^**^	6.12±0.10	5.90±0.08	CAT (µ mol H_2_O_2_/min/mg protein)
23.35±0.28^b^	14.11±0.22^**^	25.92±0.27^*^	24.39±0.47	SOD (µ/mg protein

Each values represents the mean ± S.E., n = 8. Values marked with an asterisk(*) differ significantly from control value (* P<0.05,** P<0.01), while those marked with a letter (b) differ significantly from HgCl_2_ group (** P<0.01).

### Testicular cholesterol and plasma testosterone

Mean level of testicular cholesterol significantly (P<0.01) decreased in both HgCl_2_-exposed and *S. platensis*+HgCl_2_ groups by 47% and 7%, respectively (Table 3). Moreover, the concentration of plasma testosterone was significantly (P<0.01) blunted in HgCl_2_ and *Spirulina*+HgCl_2_ groups by 41% and 17%, respectively. However, the mean values of the two parameters in *S. platensis*+HgCl_2_ group were significantly higher (P<0.01) than those of HgCl_2_ group (Table 3).

**Table 3:** Effect of *S. platensis* on testicular cholesterol and plasma testosterone concentrations in experimental HgCl_2_-exposed rats as compared to control group.

Groups				
*Spirulina*+HgCl_2_	HgCl_2_	*Spirulina*	Control	Parameters
0.28±0.003^**b^	0.16±0.002^**^	0.31±0.01	0.30±0.004	Testicular cholesterol (mg/100 g bw)
2.10±0.15^*b^	1.49±0.06^**^	2.68±0.07	2.54±0.08	Plasma testosterone (ng/ml)

Each values represents the mean ± S.E., n = 8. Values marked with an asterisk (*) differ significantly from control value (* P<0.05,** P<0.01), while those marked with a letter differ (b) significantly from HgCl_2_ group (* P<0.01).

### Weights of reproductive organ

The values of organ weights are shown in Table 4. The weights of testes and accessory sex organs decreased significantly (P<0.01) in both HgCl_2_-exposed and *S. platensis*+HgCl_2_ groups. However, the values of accessory sex organs of *S. platensis*+HgCl_2_ group were significantly higher than those of the HgCl_2-_exposed group.

**Table 4:** Effect of *S. platensis* on reproductive organ weights (g) relative to body weight in experimental HgCl_2_-exposed rats as compared to control group.

Groups				
*Spirulina*+HgCl_2_	HgCl_2_	*Spirulina*	Control	Organs
1.06±0.03**	1.07±0.02**	1.18±0.02	1.23±0.01	Testis
0.05±0.003**a	0.04±0.003**	0.06±0.005	0.07±0.003	Vas deferens
0.24±0.003b	0.21±0.007**	0.24±0.008	0.25±0.003	Epididymis
0.23±0.01**a	0.19±0.01**	0.29±0.01	0.31±0.02	Prostate
0.33±0.02**b	0.26±0.01**	0.50±0.04	0.52±0.01	Seminal vesicle

The relative organ weight was calculated by use of the formula: organ weight/body weight ×100. Each values is the mean ± S.E., n = 8. Values marked with an asterisk (*) differ significantly from control value; **P<0.01. Those marked with a letter differ significantly from HgCl_2_ group; P^a^<0.05; P^b^<0.01.

### Epididymal sperm characteristics

The results of sperm analysis are presented in Table 5. Both sperm motility and concentration of *S. platensis* group increased significantly (P<0.01) as compared to control values. Although HgCl_2_ treatment caused a significant decrease (P<0.01) in sperm motility and concentration, it resulted in a significant (P<0.01) increase in head, tail and total sperm abnormalities when compared with the values of control group. The administration of *S. platensis*+HgCl_2_ in combination tended to increase sperm motility along with sperm concentration and to decrease abnormal sperm rates as compared to HgCl_2_ group.

**Table 5:** Effect of *S. platensis* on sperm morphological quality parameters in experimental HgCl_2_-exposed rats as compared to control group.

Groups				
*Spirulina*+HgCl_2_	HgCl_2_	*Spirulina*	Control	Parameters
79.32±1.02**b	63.87±1.57**	89.08±0.47**	84.50±0.56	Sperm motility (%)
29.49±1.29b	21.69±0.84**	33.67±1.41**	27.39±0.75	Sperm count per epididymis (million/epididymis)
Abnormal sperm rate (%)+				
5.60±0.36**b	8.25±0.25**	2.42±0.22	2.11±0.13	Head
2.45±0.32a	3.40±0.24**	2.26±0.18	2.06±0.10	Tail
7.99±0.58**b	11.66±0.62**	4.68±0.27	4.17±0.19	Total

+ Normal sperms were characterized with hook-shaped head and straight tail in control animals. Morphological abnormalities observed in HgCl_2_-exposed spermatozoa were absence of head, head winding around tail, coiled tails and kinks in midpiece and tail regions, bent and curved tail. Each values is the mean ± S.E., n = 8. Values marked with an asterisk (*) differ significantly from control value: **P<0.01, those marked with letter differ significantly from HgCl_2_ group; P^a^<0.05, P^b^<0.01.

### Histopathology

**Figure 1:**
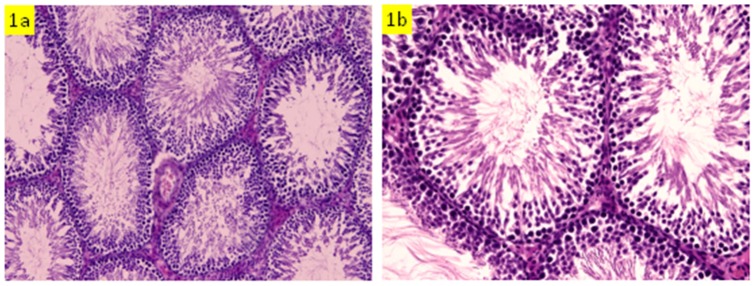
Testicular section of control rat which show normal spermatogenesis and cell arrangement in the seminiferous tubules (a: H&E x200, b: H&Ex400).

**Figure 2:**
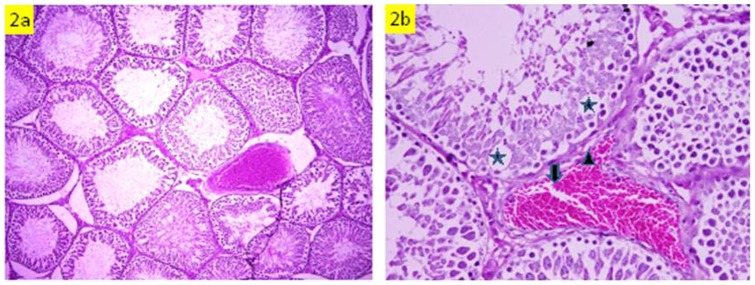
Testicular sections of HgCl_2_-exposed group. Note impaired spermatogenesis in some seminiferous tubules, degenerative areas in spermatogenic layers (Star), irregular vacuolized basement membrane (head of arrow) and hemorrhage in interstitial space (Arrow). (a: H&E ×200, b: H&Ex400).

**Figure 3:**
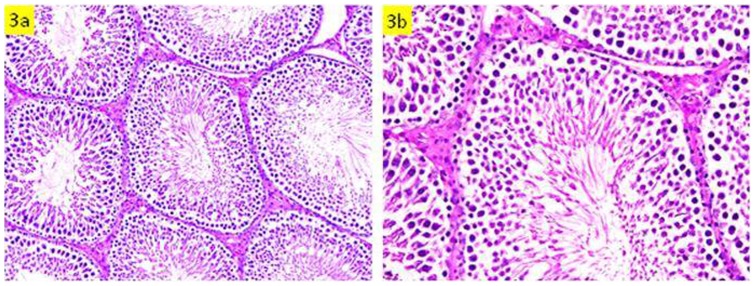
Testicular section of *Spirulina*+HgCl_2_ group which show normal spermatogenesis and cell arrangement (a: H&E ×300, b: H&E×400).

Histopathological examination revealed that rats administered with HgCl_2_ alone exhibited testicular lesions in some seminiferous tubules comprising decrease of luminal spermatozoa, irregular basement membrane, disorganization and degeneration of some spermatogenic cells and hemorrhage in interstitial tissues, as compared to the control group (Figure 1 & 2). Pre-administration of *S. platensis* protected the testicular tissue of rats exposed to HgCl_2_, as evidenced by the appearance of normal structure of testicular seminiferous tubules (Figure 3).

## Discussion

Several *in vivo* and in *vitro* studies have reported that the exposure of animals to inorganic or organic forms of mercury are accompanied by induction of oxidative stress [43] and elevation of production of reactive oxygen species (ROS) which lead to cell death [44]. Oxidative damage may result from decreased clearance of ROS by scavenging mechanisms. In the present study, HgCl2 exposure correlated with increased levels of oxidative stress and antioxidant biomarkers in the testis of rats, represented by decreased levels of GSH along with antioxidant enzymes (SOD, CAT, and GPx) and the increase of testicular MDA and blood hydroperoxide (Tables 1 & 2). MDA is one of the major products of peroxidized polyunsaturated fatty acids and increased MDA content is an important indicator of lipid peroxidation. It has been shown previously that HgCl_2_ increase MDA level in testicular tissue [12]. Treatment of rats with a combination of *Spirulina* and HgCl_2_ attenuated the HgCl_2_– induced increase in MDA level, which indicates that *Spirulina* may have a beneficial effect in reducing HgCl_2_ toxicity. Moreover, *Spirulina* reduced the accumulation of mercury in the testis. It has been reported that *Spirulina* contains vitamin E and selenium (Se) which play a crucial rule in hindering the absorption of mercury in the gastrointestinal tract [11], [45]. The most important role for Se as an essential trace element is acting as an antioxidant agent because it an essential component of the selenoprotein glutathione peroxidase (GPx). Cell membranes are protected by GPx from ROS-mediated oxidative damage resulting from the formation of hydrogen peroxide (H_2_0_2_) during normal metabolism in the cells' mitochondria. In this context, GPx converts H_2_0_2_ into water before it can produce damaging ROS. Vitamin E works in a similar way to prevent ROS-mediated membrane damage by binding up ROS within the cell membrane. In this way, Se and vitamin E act in concert to prevent damage to cell membranes, DNA and other cellular structures from damage by free radicals [46].

Various mechanisms have been proposed to explain the biological toxicity of HgCl_2_, including oxidative stress. Numerous studies on mercury toxicities have revealed that it generates ROS and has a great affinity for SH groups of biomolecules, thus depleting intracellular thiols including reduced glutathione [14]. It is postulated that the antioxidant GSH depletion by mercury may be a trigger for the production of reactive oxygen species (ROS) that induce lipid, protein, and DNA oxidation. Generation of ROS in the cytoplasm of cells may increase the hydrogen peroxide production and lipid peroxidation of mitochondrial membrane, resulting in loss of membrane integrity and finally cell necrosis or apoptosis [47]–[49]. In the present investigation, Mercury administration depleted GSH content in testicular tissues that made spermatogenic cells more susceptible to oxidative damage, especially during increased free radical production. GSH is an important intracellular antioxidant that spontaneously neutralizes several reactive oxygen species [50]. In the present investigation, *Spirulina* elevated testicular content of GSH. It has been reported that GSH, both as a carrier of mercury and an antioxidant agent, has specific roles in protecting the body from mercury toxicity. Glutathione, specifically binds with methyl mercury, forms a complex that prevents mercury from binding to cellular proteins and causing damage to both enzymes and tissue [51]. Glutathione–mercury complexes also reduce intracellular damage by preventing mercury from entering tissue and cells, and becoming an intracellular toxin [51].

Cells have a number of mechanisms to protect themselves from the toxic effect of ROS. SOD removes superoxide radical by converting it into H_2_O_2_ which is rapidly converted to water by CAT or GPx. Moreover, GPx reduces lipid hydroperoxides to alcohols. Therefore, any alteration in the activity of these enzymes may result in a number of deleterious effects due to accumulation of superoxide radicals and hydrogen peroxide. In the present study, the activities of antioxidant enzymes, SOD, CAT, and GPx in testicular tissues were concomitantly restored to normal level by *Spirulina* supplementation to HgCl_2_, which is indicative of the ROS-scavenging role that *Spirulina* could play whereby lessening tissue damage and subsequently improving the activities of these antioxidant enzymes. In *Spirulina*+HgCl_2_ group, *Spirulina* ameliorated the antioxidant status of testicular tissues in terms of increased GSH and antioxidant enzymes with decreased MDA contents. Hence, it could be concluded that *Spirulina* might have a potential role in preventing Hg-Cl_2_-induced testicular injuries. It could also be proposed that the beneficial effects of *Spirulina* may be due to its antioxidant properties that could revive endogenous cellular antioxidant defense system. *Spirulina* is considered a valuable additional food source of some macro- and micro-nutrients including high-quality protein, iron, gamma-linolenic fatty acids, carotenoids and vitamins [52]. The metalloprotective role of *Spirulina* may be attributed to the presence of potent antioxidant components as β-carotene, vitamin C, E, selenium and brilliant blue polypeptide pigment phycocyanin. It has been reported that β-carotene of *Spirulina* may reduce cell damage, especially the damage to DNA molecules, thus playing the role in the repair of regeneration process of damaged cells [53]. β- Carotene of *Spirulina* may scavenge free radicals generated by HgCl_2_, thus reducing lipid peroxidation. The antioxidant mechanism of β -carotene has been suggested to be occur *via* quenching of singlet oxygen, free radical scavenging and chain breaking during lipid peroxidation [54]. Vitamin E prevents lipid peroxidation and maintains GSH and ascorbic acid levels in damaged tissue by inhibiting free radical formation [55]. Moreover, these findings have been recently supported by Al-Attar [56] who showed that the administration of vitamin E protects against heavy metals-induced renal and testicular oxidative stress and injuries. Vitamin C reduces the chemical toxicity by decreasing the covalent binding of reactive intermediates and eliminating free radical metabolites [57]. Selenium present in *Spirulina* induces the selenium-containing enzyme GPx, proteins or compounds such as selenodiglutathione, selenocysteine and dimethylselenide, which are known to modulate the toxic effects of heavy metals [58]. It has been reported that phycocyanin content of *Spirulina* significantly inhibited peroxyl radical-induced lipid peroxidation [59].

Concerning the hormone level, a fall in the plasma testosterone level in the animals treated with mercury was observed (Table 3). The decrease in the testosterone level seems to be due to a reduction in the activity of enzymes involved in the biosynthesis of testosterone [60] or due to the decrease in testicular cholesterol, a precursor of testosterone synthesis. After mercury treatment, sperm concentration in the epididymis decreased (Table 5), perhaps due to a low level of sperm production in the testis, which could be related to a low level of testosterone, a prime regulator for sperm production [61]. As growth of accessory sex glands requires testosterone [62], the decrease in the weight of these glands due to mercury treatment can result from the reduction in the testosterone level (Table 4). The decrease in epididymal sperm concentration is consistent with histologic examination of the seminiferous tubules, which showed a decrease in luminal spermatozoa in HgCl_2_-exposed rats (Figure 2). In the present investigation, reduction in sperm number/epididymis weight and motility was associated with an increase of sperm abnormalities in rats exposed to HgCl_2_, which suggests the HgCl_2_ may interfere with spermatogenesis by crossing the blood – testis barrier and gaining access to germinal cells. The adverse effects of HgCl_2_ on mammalian testicular tissue have been reported with marked testicular spermatogenic degeneration at the spermatocyte level in rats [7]. The spermatozoa membranes are rich in polyunsaturated fatty acids, so they are susceptible to ROS attack and lipid peroxidation [63] as a result of exposure to mercury. Lipid peroxidation reaction causes membrane damage which leads to a decrease in sperm motility, presumably by a rapid loss of intracellular ATP, and an increase in sperm morphology defects [64], [65]. Mercury compounds have been reported to cause DNA breaks by means of free-radical mediated reactions [44] that may cause the increase in the frequency of spermatozoa with abnormal heads. Several active components in *Spirulina* [54], [55], [59] may scavenge ROS generated by mercury, reduce lipid peroxidation and enhance the activity of antioxidant enzymes whereby leading to protection against mercury-induced testicular damages which are manifested by an increase in sperm abnormalities and fall in testosterone level. Thus, the antioxidative properties of *Spirulina* may play a positive role in the defense against oxidative stress induced by HgCl_2_.

It has been observed that the addition of *S. platensis* to a diet in boars leads to an increase in sperm concentration, viability and lactate dehydrogenase activity, which has been correlated with sperm motility [44]. The present investigation clearly demonstrated that the treatment of rats with *S. platensis* positively impact upon sperm quality parameters, as manifested by an increase of sperm motility and count. The improvement of sperm quality may be due to the antioxidant components of *Spirulina*, such as α – tochopherol (vitamin E), ascorbic acid (vitamin C) and selenium that improve testicular functions and sperm quality [66], [67]. Vitamin C is a well-known antioxidant that is present in the testis protecting it from oxidative damage [68]. Thus, it has been recently reported that the decrease in the testicular level of vitamin C are correlated with methylparathion-mediated effects on sperm quality and count in rats [42].

Moreover, ß-carotene, a component of *Spirulina*, has been reported to enhance reproductive functions and growth in mice [69]. Moreover, scientific studies have shown that the synergistic action exhibited by a broad spectrum of antioxidants is more efficient than the activity of a single antioxidant. In addition, antioxidants from natural sources (primarily food) have a higher bioavailability and, therefore, higher protective efficacy than the synthetic ones [70].

In conclusion, the study demonstrates that *S. platensis* may protect testes against HgCl_2_-induced testicular damage, as evidenced by its effective restoration potency of oxidative stress markers, activities of enzymatic antioxidant biomarkers and histopathological alterations back to control level. Hence, it might be postulated that the protection observed by *Spirulina* could be attributed to its rich antioxidants content whereby scavenging HgCl_2_-induced ROS.

## References

[1] Allouche L, Hamadouche M, Touabti A (2009) Chronic effects of low lead levels on sperm quality, gonadotropins and testosterone in albino rats. Exp Toxicol Pathol 61: 503–510. 19188052 10.1016/j.etp.2008.12.003

[2] Gunn S, Gould T (1970) Cadmium and other mineral elements. In: Johnson AD, Gomes WR, Vademark NL, editors. The testis, vol.111. Influencing factors. New York: Academic Press p. 378–481.

[3] Anderson M, Pedigo N, Katz R, George W (1992) Histopathology of testis from mice chronically treated with cobalt. Reprod Toxicol 6: 41–50. 1562797 10.1016/0890-6238(92)90019-p

[4] Ekstrand J, Bjorkman L, Edlund C, Sandborgh E (1998) Toxicological aspects on the release and systemic uptake of mercury from dental amalgam. Euro J Oral Sci 106: 678–686. 10.1046/j.0909-8836.1998.eos10602ii03.x 9584901

[5] Fossato da Silva D, Teixeira C, Scarano W, Favareto A, Fernandez C, et al (2011) Effects of methyl mercury on male reproductive functions in Wistar rats. Reprod Toxicol 31: 431–439. 21262343 10.1016/j.reprotox.2011.01.002

[6] Khan A, Atkinson A, Graham T, Thompson S, Ali S, et al (2004) Effects of inorganic mercury on reproductive performance of mice. Food Chem Toxicol 42: 571–577. 15019180 10.1016/j.fct.2003.10.018

[7] Vachhrajani K, Makhija S, Chinoy N, Chowdhury A (1988) Structural and functional alterations in testis of rats after mercuric chloride treatment. J Reprod Bio Compara Endocrinol 8: 97–104.

[8] Rao M (1989) Histophysiological changes of sex organs in methyl mercury intoxicated mice. Endocrinologia Experimentalis 23: 60–65. 2714228

[9] Mohamed M, Burbacher T, Mottet M (1987) Effects of methyl mercury on testicular functions in *Macaca fascicularis* monkey. Pharmacol Toxicol 60: 29–36. 3562387 10.1111/j.1600-0773.1987.tb01715.x

[10] Rao M, Gangadharan B (2008) Antioxidative potential of melatonin against mercury induced intoxication in spermatozoa in vitro. Toxicol in vitro 22: 935–942. 18329842 10.1016/j.tiv.2008.01.014

[11] Su L, Wang M, Yin S, Wang H, Chen L, et al (2008) The interaction of selenium and mercury in the accumulations and oxidative stress of rat tissues. Ecotoxicol Environ Safety 70: 483–489. 17644179 10.1016/j.ecoenv.2007.05.018

[12] Boujbiha MA, Hamden K, Guermazi F, Bouslama A, Omezzine A, et al (2009) Testicular toxicity in mercuric chloride treated rats: Association with oxidative stress. Reprod Toxicol 28: 81–89. 19427169 10.1016/j.reprotox.2009.03.011

[13] Lorschieder FL, Vimy MJ, Summers AO (1995) Mercury exposure from “silver” tooth filling: emerging evidence questions a traditional dental paradigm. FASEB J 9: 504–508. 7737458

[14] Hansen JM, Zhang H, Hones DP (2006) Differential oxidation of thio-redoxin-1, thioredoxin-2, and glutathione by metal ions. Free Radic Biol Med 40: 138–145. 16337887 10.1016/j.freeradbiomed.2005.09.023

[15] Valko M, Morris H, Cronin MTD (2005) Metals, toxicity and oxidative stress. Curr Med Chem 12: 161–208. 10.2174/0929867053764635 15892631

[16] Agarwal A, Saleh RA, Bedaiwy MA (2003) Role of reactive oxygen species in the pathophysiology of human reproduction. Fertil Steril 79: 829–43. 12749418 10.1016/s0015-0282(02)04948-8

[17] Diemer T, Allen JA, Hales KH, Hales DB (2003) Reactive oxygen disrupts mitochondria in MA-10 tumor Leydig cells and inhibits steroidogenic acute regulatory (StAR) protein and steroidogenesis. Endocrinol 144: 2882–2891. 10.1210/en.2002-0090 12810543

[18] Popescu HI (1978) Poisoning with alkyl mercuric compounds. Br Med J 1: 23–47.620134

[19] Keck C, Bergman M, Ernst E, Muller C, Kliensch S, et al (1993) Autometallographic detection of mercury in testicular tissue of infertile men exposed to mercury vapour. Reprod Toxicol 7: 469–75. 8274823 10.1016/0890-6238(93)90092-l

[20] Cohen Z (1997) The chemicals of Spirulina, In: Vonshak, A. (Ed), *Spirulina platensis* (Arthrospira): Physiology, Cell-biology and Biotechnology, Taylor and francis Ltd, 175–204.

[21] Peter C (2008) Antioxidant potential of *Spirulina platensis* preparations. Phytotherapy Res 22: 627–33. 10.1002/ptr.2310 18398928

[22] Karadeniz A, Yildiri A, Simsek N, Kalkan Y, Celebi F (2008) *Spirulina platensis* protects against gentamicin-induced nephrotoxicity in rats. Phytotherapy Res 22: 1506–1510. 10.1002/ptr.2522 18690652

[23] Torress-Duran PV, Ferreira-Hermosillo A, Juarez-Oropeza MA (2007) Antihyperlipemic and antihypertensive effects of *Spirulina maxima* in an open sample of Mexican population: a preliminary report. Lipids health disease 6: 33–41. 10.1186/1476-511X-6-33 PMC2211748 18039384

[24] Sharma MK, Sharma A, Kumar A, Kumar M (2007) *Spirulina fusiforms* provides protection against mercuric chloride induced oxidative stress in Swiss albino mice. Food Chem Toxicol 45: 2412–2419. 17706852 10.1016/j.fct.2007.06.023

[25] Romay C, Armesto J, Remikrez D, Gonzalez R, Ledon L, et al (1998) Antioxidant and anti-inflammatory properties of c-phycocyanin from blue-green algae. Inflamm Res 47: 36–41. 9495584 10.1007/s000110050256

[26] Salazar M, Martinez E, Madrigal E, Ruiz LE, Chamorro GA (1998) Subchronic toxicity in mice fed *Spirulina maxima.* . J Ethenopharmacol 62: 235–241. 10.1016/s0378-8741(98)00080-4 9849634

[27] Salazar M, Chamorro GA, Salazar S, Steele CE (1996) Effect of *Spirulina maxima* consumption on reproduction and peri- and postnatal development in rats. Food Chem Toxicol 34: 353–359. 8641661 10.1016/0278-6915(96)00000-2

[28] Chamorro GA, Salazar M, Pages N (1996) Dominant lethal study of *Spirulina maxima* in male and female rats after short term feeding. Phytotherapy Res 10: 28–32.

[29] Saxena PS, Kumar M (2004) Modulatory potential of *Spirulina fusiformis* on testicular phosphatases in Swiss albino mice against mercury intoxication. Indian J Exp Biol 42: 998–1002. 15511004

[30] Simsek N, Karadeniz A, Kalkan Y, Keles ON, Unal B (2009) *Spirulina platensis* feeding inhibited the anemia and leucopenia-induced lead and cadmium in rats. J Hazar Mat 164: 1304–1309. 10.1016/j.jhazmat.2008.09.041 18976856

[31] Peixoto NC, Roza T, Flores EMM, Pereira ME (2003) Effects of zinc and cadmium on HgCl_2_-δ-ALA-D inhibition and Hg levels in tissues of suckling rats. Toxicol Lett 146: 17–25. 14615064 10.1016/j.toxlet.2003.08.006

[32] Mathur N, Pandey G, Jain GC (2010) Male Reproductive Toxicity of Some Selected Metals: A Review. J Biol Sci 10: 396–404.

[33] Ohkawa H, Ohishi N, Yagi K (1979) Assay of lipid peroxides in animal tissues by thiobarbituric acid reaction. Analyt Biochem 95: 351–358. 36810 10.1016/0003-2697(79)90738-3

[34] Aboul-Soud MAM, Al-Othman AM, El-Desoky GE, Al-Othman ZA, Yusuf K, et al (2011) Hepatoprotective effects of vitamin E/selenium against malathion-induced injuries on the antioxidant status and apoptosis-related gene expression in rats. J Toxicol Sci 36: 285–296. 21628957 10.2131/jts.36.285

[35] Shinyashiki M, Kumagai Y, Nakajima H, Nagafune J, Homma-Takeda S, et al (1998) Differential changes in rat brain nitric oxide synthase in vivo and *in vitro* by methylmercury. Brain Res 798: 147–155. 9666107 10.1016/s0006-8993(98)00400-4

[36] Moron MS, Depierre JW, Mannervik B (1979) Levels of GSH, GR and GST activities in rat lung and liver. Biochimica et Biophysica Acta 582: 67–78. 760819 10.1016/0304-4165(79)90289-7

[37] Asada K, Takahashi M, Nagate M (1974) Assay and inhibitors of spinach superoxide dismutase. Agric Biol Chem 38: 471–473.

[38] Aebi H (1984) Catalase *in vitro* . Method Enzymol 105: 121–126. 10.1016/s0076-6879(84)05016-3 6727660

[39] Paglia DE, Valentine WN (1967) Studies on the quantitative and qualitative characterization of erythrocyte glutathione peroxidase. J Lab Clin Med 70: 158–169. 6066618

[40] Zlatki S, Azaki B, Boyle GJ (1953) In:Harold V, editor. Practical clinical biochemistry. London: Arnol Heinemann Publication.

[41] Narayana K, D'Souza U, Rao K (2002) Ribavirin induced sperm shape abnormalities in Wistar rat. Mutat Res 513: 193–196. 11719104 10.1016/s1383-5718(01)00308-4

[42] Narayana K, Prashanthi N, Nayanatar A, Kumar HHC, Abhilash K, et al (2005) Effects of methyl parathion (o,o-dimethyl o-4-nitropheny phosphorothioate) on rat sperm morphology and sperm count, but not fertility, are associated with decreased ascorbic acid level in the testis. Mutat Res 588: 28–34. 16226487 10.1016/j.mrgentox.2005.08.012

[43] Nogueira CW, Soares FA, Nascimento PC, Muller D, Rocha JBT (2003) 2, 3- Dimercaptopropane -1-sulfonic acid and meso-2,3-dimercaptosuccinic acid increase mercury- and cadmium-induced inhibition of σ-amino levulinate dehydratase. Toxicol 184, 85–95. 10.1016/s0300-483x(02)00575-9 12499112

[44] Park E-J, Park K (2007) Induction of reactive oxygen species and apoptosis in BEAS-2B cells by mercuric chloride. Toxicol In Vitro 21: 789–790. 17363214 10.1016/j.tiv.2007.01.019

[45] Rao MV, Sharma PSN (2001) Protective effect of Vitamin E against mercuric chloride reproductive toxicity in male mice. Reprod Toxicol 15: 705–712. 11738524 10.1016/s0890-6238(01)00183-6

[46] Al-Othman AM, Al-Numair KS, El-Desoky GE, Yusuf K, Al-Othman ZA, et al (2011) Protection of α -tocopherol and selenium against acute effects of malathion on liver and kidney of rats. Afr J Pharm Pharmaco 10: 1263–1271.

[47] Jezek P, Hlavata L (2005) Mitochondria in homeostasis of reactive oxygen species in cell, tissues, and organism. Int J Biochem Cell Biol 37: 2478–503. 16103002 10.1016/j.biocel.2005.05.013

[48] Kaur P, Aschner M, Syversen T (2006) Glutathione modulation influences methyl mercury induced neurotoxicity in primary cell cultures of neurons and astrocytes. Neurotoxicol 2:, 492–500. 10.1016/j.neuro.2006.01.010 16513172

[49] Oh IS, Datar S, Koch CJ, Shapiro IM, Shenker BJ (1997) Mercuric compounds inhibit human monocyte function by inducing apoptosis: Evidence for formation of reactive oxygen species, development of mitochondrial membrane permeability transition and loss of reductive reserve. Toxicol 124: 211–224. 10.1016/s0300-483x(97)00153-4 9482123

[50] Lu SC (1999) Regulation of hepatic glutathione synthesis: current concepts and controversies. FASEB J 1:, 1169–1175. 10385608

[51] Kromidas L, Trombetta LD, Jamall IS (1990) The protective effects of glutathione against methyl mercury cytotoxicity. Toxicol Let 51: 67–80. 2315960 10.1016/0378-4274(90)90226-c

[52] Mazo VK, Gmoshinskii IV, Zilova IS (2004) Microalgae *Spirulina* in human nutrition. Voprosy Pitaniia 73: 45–53. 15049159

[53] Luxia AS, Monica S, Ornella C, Plizzala B, Laura R, et al (1996) Effect of b-carotene on cell cycle progression of human fibroblasts. Mutagenesis 17: 2395–2401. 10.1093/carcin/17.11.2395 8968054

[54] Gerster H (1993) Anticarcinogenic effect of common carotenoids. Inter J for Vit Nut Res 63: 93–121. 8407171

[55] Garg MC, Chaudhary DP, Bansal DD (2005) Effect of vitamin E supplementation on diabetes induced oxidative stress in experimental diabetes in rats. Ind J of Exp Bio 43: 177–80. 15782820

[56] Al-Attar AM (2011) Antioxidant effect of vitamin E treatment on some heavy metals-induced renal and testicular injuries in male mice. Saudi J Biol Sci 18, 63–72. 10.1016/j.sjbs.2010.10.004 PMC3730955 23961105

[57] Traber MG, Stevens JF (2011) Vitamins C and E: Beneficial effects from a mechanistic perspective. Free Radic Biol Med 51: 1000–1013. 21664268 10.1016/j.freeradbiomed.2011.05.017 PMC3156342

[58] El-Demerdash FM (2001) Effects of selenium and mercury on the enzymatic activities and lipid peroxidation in brain, liver and blood of rats. J Environ Sci Health, Part-B 36: 489–99. 10.1081/PFC-100104191 11495025

[59] Bhat VB, Madyastha KM (2000) C-phycocyanin: a potent peroxyl radical scavenger *in vivo* and *in vitro* . Biochem Biophy Res Comm 275: 20–25. 10.1006/bbrc.2000.3270 10944434

[60] McVey MJ, Cooke GM, Curran IH, Chan HM, Kubow S, et al (2008) An investigation of the effects of methyl mercury in rats fed different dietary fats and proteins: testicular steroidogenic enzymes and serum testosterone levels. Food Chem Toxicol 46: 270–279. 17869401 10.1016/j.fct.2007.08.004

[61] Steinberger E (1975) Hormonal regulation of the seminiferous tubule function. In: French FS, Hansson V, Ritzen EM, Neyfeh SN, editors. Hormonal regulation of spermatogenesis. New York: Plenum Press p. 337–352.

[62] Barkeley MS, Goldmass BD (1977) A quantitative study of serum testosterone. Sex accessory organ growth and the development of inter male aggression in the mouse. Horm Behav 8: 208–218. 558957 10.1016/0018-506x(77)90038-1

[63] Mandava V, Rao BG (2008) Antioxidative potential of melatonin against mercury induced intoxication in spermatozoa *in vitro* . Toxicol In Vitro 22: 935–942. 18329842 10.1016/j.tiv.2008.01.014

[64] De Lamirande, E, Gagnon C (1992) Reactive oxygen species and human spermatozoa. I. Effects on the motility of intact spermatozoa and on sperm axonemes; and II. Depletion of adenosine triphosphate plays an important role in the inhibition of sperm motility. J Androl 13: 368–386. 1331007

[65] Kistanova E, Marchev Y, Nedeva R, Kacheva D, Shumkov K, et al (2009) Effect of the *Spirulina platensis* induced in the main diet on boar sperm quality. Biotech animal husband 25(5–6): 547–57.

[66] Mohammadi S, Movahedin M, Mowla SJ (2008) Antioxidant effects of selenium on sperm parameters and testicular structure in young and aged mice. J reprod. Infertility 9: 229–237.

[67] Yousef MI, Abdallah GA, Kamel KI (2003) Effect of ascorbic acid and vitamin E supplementation on semen quality and biochemical parameters of male rabbits. Animal Reprod Sci 76: 99–111. 10.1016/s0378-4320(02)00226-9 12559724

[68] Sonmez M, Turk G, Yuce A (2005) The effect of ascorbic acid supplementation on sperm quality, lipid peroxidation and testosterone levels of male Wistar rats. Theriogenology 63: 2063–2072. 15823361 10.1016/j.theriogenology.2004.10.003

[69] Nagasawa H, Konishi R, Yamamoto K, Ben-Amotz A (1989) Effects of beta-carotene-rich algae on reproduction and body growth in mice. In Vivo 3: 79–81. 2519842

[70] Gey KF (1998) Vitamins E plus C and interacting co-nutrients required for optimal health. A critical and constructive review of epidemiology and supplementation data regarding cardiovascular disease and cancer. Biofactors 7: 113–174. 9523035 10.1002/biof.5520070115

